# Poly-ε-Caprolactone/Fibrin-Alginate Scaffold: A New Pro-Angiogenic Composite Biomaterial for the Treatment of Bone Defects

**DOI:** 10.3390/polym13193399

**Published:** 2021-10-02

**Authors:** Jiongyu Ren, Nupur Kohli, Vaibhav Sharma, Taleen Shakouri, Zalike Keskin-Erdogan, Siamak Saifzadeh, Gary I. Brierly, Jonathan C. Knowles, Maria A. Woodruff, Elena García-Gareta

**Affiliations:** 1 Faculty of Engineering, Queensland University of Technology, Brisbane, QLD 4059, Australia; edward.ren@qut.edu.au (J.R.); gibrierly@gmail.com (G.I.B.); mia.woodruff@qut.edu.au (M.A.W.); 2 Regenerative Biomaterials Group, The RAFT Institute & The Griffin Institute, Northwick Park & Saint Mark’s Hospital, London HA1 3UJ, UK; n.kohli@imperial.ac.uk (N.K.); sharmav@raft.ac.uk (V.S.); 3 Department of Mechanical Engineering, Imperial College London, London SW7 1AL, UK; 4 Division of Biomaterials & Tissue Engineering, Eastman Dental Institute, University College London, Rowland Hill Street, London NW3 2PF, UK; taleen.shakouri.18@ucl.ac.uk (T.S.); zalike.keskin.17@ucl.ac.uk (Z.K.-E.); j.knowles@ucl.ac.uk (J.C.K.); 5 Medical Engineering Research Facility, Queensland University of Technology, Brisbane, QLD 4059, Australia; siamak.saifzadeh@qut.edu.au; 6 UCL Eastman-Korea Dental Medicine Innovation Centre, Dankook University, Cheonan 31116, Korea; 7 Department of Nanobiomedical Science & BK21 PLUS NBM Global Research Centre for Regenerative Medicine, Dankook University, Cheonan 31116, Korea

**Keywords:** bone regeneration, angiogenesis, polycaprolactone, fibrin, alginate

## Abstract

We hypothesized that a composite of 3D porous melt-electrowritten poly-ɛ-caprolactone (PCL) coated throughout with a porous and slowly biodegradable fibrin/alginate (FA) matrix would accelerate bone repair due to its angiogenic potential. Scanning electron microscopy showed that the open pore structure of the FA matrix was maintained in the PCL/FA composites. Fourier transform infrared spectroscopy and differential scanning calorimetry showed complete coverage of the PCL fibres by FA, and the PCL/FA crystallinity was decreased compared with PCL. In vitro cell work with osteoprogenitor cells showed that they preferentially bound to the FA component and proliferated on all scaffolds over 28 days. A chorioallantoic membrane assay showed more blood vessel infiltration into FA and PCL/FA compared with PCL, and a significantly higher number of bifurcation points for PCL/FA compared with both FA and PCL. Implantation into a rat cranial defect model followed by microcomputed tomography, histology, and immunohistochemistry after 4- and 12-weeks post operation showed fast early bone formation at week 4, with significantly higher bone formation for FA and PCL/FA compared with PCL. However, this phenomenon was not extrapolated to week 12. Therefore, for long-term bone regeneration, tuning of FA degradation to ensure syncing with new bone formation is likely necessary.

## 1. Introduction

The treatment of bone defects caused by trauma, infection, tumour resection, or inherent genetic disorders is a clinical challenge. Current surgical methods often involve grafting of either autologous, the “gold standard”, or allogenic bone, but they are subject to limitations, such as limited bone supply and donor site morbidity in the case of autologous bone graft or disease transmission, and fracture and non-union in the case of allogenic bone grafting [1]. Xenogenic bone is another source of bone tissue with both osteoconductive and osteoinductive properties. However, a graft as similar as possible to the host bone in terms of microstructure and composition is recommended for optimum biological behaviour and clinical performance [2]. Implants made of metals such as titanium and stainless steel are also used to treat bone defects; however, they have a limited lifespan and cause stress shielding due to excessive material strength [3]. These disadvantages are driving the development of new biomaterials that can act as efficacious bone graft substitutes. 

Natural bone from human or animal sources serves as the model for the development of bone graft substitutes, where materials of natural and/or synthetic origin can be combined with therapeutic cells and factors [4]. The different materials used are natural and synthetic biodegradable polymers [5,6], ceramics [7], metals [8], carbon-based materials [9], and composites, which are a combination of two or more materials [1,6,10]. Particularly, synthetic polymers have been extensively used to create 3D structures for bone regeneration due to the good biocompatibility, cost-effectiveness, and versatility. One of these synthetic polymers is poly-ε-caprolactone (PCL), which is a hydrophobic, semi-crystalline polyester that shows good solubility and a low melting point (59–64 °C). PCL has superior rheological properties over many of its resorbable polymer counterparts, which means that it can be manufactured and manipulated into a large range of shapes and structures [11]. These properties have allowed PCL to be manufactured into scaffolds via 3D printing, electrospinning, and melt-electrowriting (MEW) [12,13,14,15,16]. 

As a Food and Drug Administration (FDA)-approved polymer, PCL shows excellent biocompatibility and slow degradation via hydrolysis [11]. However, the hydrophobic PCL surface is not ideal for cell attachment and proliferation, often requiring surface treatments to make it more hydrophilic [17]. Another disadvantage of PCL is its lack of bone forming properties. Therefore, several research groups have combined PCL with osteogenic components such as ceramics or bone morphogenetic proteins (BMPs) to promote osteogenesis [3,12,18,19,20,21,22]. Finally, PCL does not promote formation of blood vessels in vivo, which is essential for bone regeneration [23]. 

Compared with PCL, fibrin is a naturally occurring biodegradable polymer that shows pro-angiogenesis, biocompatibility, and bioactivity [24]. Unfortunately, its mechanical and biodegradable properties are very poor, thus limiting its use as a standalone scaffold for tissue engineering applications. Fibrin in combination with other materials has been used as a bio-scaffold for the regeneration of tissues such as bone, cartilage, skin, tendons, ligaments, and liver or cardiac tissue, showing a great potential for tissue regeneration [24]. In bone, fibrin has been combined with synthetic polymers to create composite bone graft substitutes that may or may not include a ceramic filler [25,26]. Invariably, the fibrin used is a simple combination of fibrinogen and thrombin, i.e., fibrin glue or fibrin sealant, applied to the PCL scaffold at the time of implantation [27,28,29,30]. 

As mentioned above, fast biodegradability and poor mechanical properties limit the use of fibrin in isolation, which is usually applied in the form of a gel [24]. Alginate is a biocompatible natural polymer with tuneable mechanical properties through cross-linking with divalent ions such as Ca^2+^ [31]. A porous, cross-linked fibrin/alginate scaffold has been developed in our laboratory for the treatment of full thickness skin wounds and is currently undergoing clinical trials [32,33,34,35]. Producing a fibrin-based scaffold rather than a gel has advantages in terms of retarding degradation and improving cell ingress into the scaffold, thus enhancing tissue regeneration and establishing a blood supply [32,33].

As explained, the treatment of bone defects still presents a significant clinical challenge, and one of the key aspects in bone regeneration, angiogenesis, is often missing from scaffold design and development. Therefore, the aim of this study was to develop a new pro-angiogenic composite biomaterial for the treatment of bone defects by using 3D porous melt-electrowritten PCL scaffolds coated throughout with a porous, cross-linked, and slowly biodegradable fibrin/alginate matrix. The hypothesis was that the combination of fibrin/alginate with a robust, resorbable PCL scaffold would provide a novel superior composite scaffold that facilitates accelerated bone regeneration due to its angiogenic potential. The novelty of our work is the introduction of a pro-angiogenic fibrin-based biomaterial component (fibrin/alginate) as part of the scaffold rather than added as a glue at the time of implantation.

## 2. Materials and Methods

### 2.1. Melt-Electrowritten (MEW) PCL 

**Figure 1:**
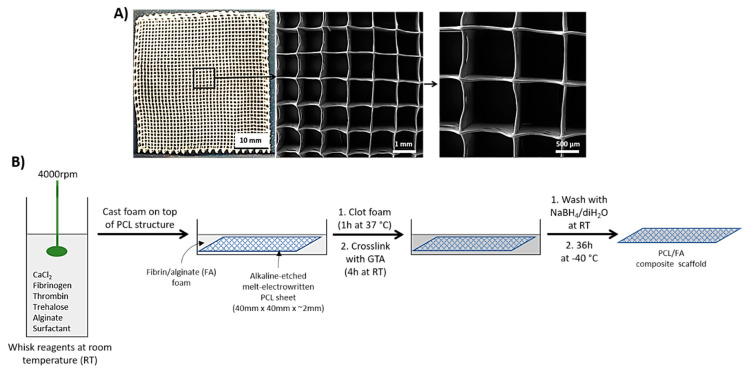
(**A**) MEW 3D PCL scaffolds used in this study. Left, representative macroscopic image of the scaffold structure. Right, representative SEM of the 3D PCL structures. (**B**) Workflow of the PCL/FA composite scaffolds’ manufacturing.

MEW PCL (Capa 6430^®^, Perstop UK Ltd., Warrington, UK) scaffolds were fabricated as described previously [14,15,16]. Scaffold sheets (40 mm × 40 mm × ~2 mm) were fabricated to have a stacked (~50 layers) 90° cross-hatched microarchitecture, with a fibre diameter of 50 μm and spacing of 1 mm (Figure 1A).

### 2.2. PCL Surface Treatment

Alkaline etching with 5M NaOH for 1 or 5 h was investigated to make PCL hydrophilic. For this purpose, 6 mm diameter discs of PCL (Capa 6430^®^, Perstop UK Limited, Warrington, UK) were used. 

An immuno-based assay [36] was used to assess fibrinogen (precursor of fibrin) adsorption onto the different PCL surfaces. Bovine fibrinogen (F8630, Sigma-Aldrich, Gillingham, UK) was reconstituted to 5 mg/mL in PBS. All available binding sites in a 96-well ELISA plate (655061, Greiner Hi-binding 96-well ELISA plate, Greiner Bio One Ltd., Stonehouse, UK) were blocked with blocking buffer (BB) (1% bovine serum albumin (BSA, A9647, Sigma-Aldrich, Gillingham, UK) in PBS) (1 h, 37 °C, 100 µL/well). All wells were washed twice with PBS (100 µL), and discs were placed at the bottom of the wells. Empty wells used as a reference were not pre-blocked. Fibrinogen (100 µL) was adsorbed over the range 0–250 µg/mL on empty wells and discs for 1 h at 37 °C in triplicate, and the excess protein was washed off with PBS. All wells were further blocked with BB (100 µL/well) for 1 h at 37 °C, followed by a PBS wash. All wells were incubated (1 h, 37 °C) first with anti-fibrinogen polyclonal antibody produced in goat (F8512, Sigma-Aldrich, St. Louis, MO, USA, 1:2000 dilution in BB) and then with anti-goat IgG alkaline phosphatase conjugate (A4187, Sigma-Aldrich, St. Louis, MO, USA, 1:1000 dilution in BB). An amount of 200 µL of wash buffer (Tris-NaCl, 0.1% BSA, 10 mM NaN_3_ and 0.1% Triton X-100 (30632GN, BDH chemicals, Poole, UK)) was added to the wells between each antibody incubation. Para-nitro-phenylphosphate (1 mg/mL in 0.1 M diethanolamine) was the substrate used to detect the phosphatase activity in the dark before stopping with 100 µL of 0.1 M EDTA (pH 9.6, 100935V, BDH chemicals, Poole, UK). An amount of 100 µL of the solution from all wells containing discs was pipetted into fresh wells, and the absorbance was read at 405 nm (Bio-Rad 550 96-well plate spectrophotometer, Bio-Rad, Watford, UK).

### 2.3. Casting of Fibrin/Alginate Matrix through the 3D PCL Structure 

Reagents needed for manufacturing the fibrin/alginate (FA) matrix (Patent ID: WO2013164635 A1) were whisked into a white foam that was cast on top of the alkaline-etched MEW 3D PCL structures. The foam was allowed to clot for 1 h at 37 °C before chemical crosslinking with 0.2% vol/vol glutaraldehyde (G6257, Sigma-Aldrich, Gillingham, UK) in 80% ethanol/20% MES (2-(N-morpholino ethanesulfonic acid (69889, Sigma-Aldrich, Gillingham, UK, 0.1 M, pH = 7.4) buffer for 4 h at room temperature. The scaffolds were washed with 0.1% wt/vol sodium borohydride (452882, Sigma-Aldrich, Gillingham, UK) in diH_2_O and diH_2_O at room temperature before lyophilisation for 36 h at –40 °C (Virtis Genesis Freeze Dryer, Biopharma, Winchester, UK) (Figure 1B).

### 2.4. Scanning Electron Microscopy (SEM)

Scaffolds (PCL, FA, and PCL/FA) were cut into 5 mm × 5 mm square pieces, placed on stubs, carbon sputter-coated, and visualised under SEM (FEI Inspect F, Oxford Instruments, Oxford, UK).

### 2.5. Attenuated Total Reflectance Fourier Transform Infrared Spectroscopy (ATR-FTIR) 

The chemical structures of the scaffolds were assessed by ATR-FTIR (System 2000, PerkinElmer, Seer Green, UK) over a range of 4000–400 cm^−1^ with a resolution of 4 cm^−1^ and presented in spectra with the area of interest of 3000–500 cm^−1^. The absorption peaks and frequencies of specific chemical groups were detected by FTIR.

### 2.6. Differential Scanning Calorimetry (DSC)

DSC thermograms were obtained using a DSC25 instrument (TA-Instruments, New Castle, DE, USA). The specimens (~2 mg each) were placed in an aluminium pan and sealed with Tzero lids and tested with a heating range of –70 to 400 °C at a rate of 10 °C/min. The melting temperature (*T*_m_) and melting enthalpy (Δ*H*) of the pure PCL, both before and after alkaline etching, and pure FA and PCL/FA were determined from the heating scan. Percentage crystallinity of PCL, with and without alkaline etching, and PCL/FA was calculated over melting peak integration, considering that the enthalpy of 100% crystalline PCL is 139.5 J/g, as indicated in Δ*H*_ref_ [37].

Crystallinity (%) = (Δ*H*_sample_/Δ*H*_reference_)∗100.

### 2.7. Cell Seeding

Scaffolds (5 mm × 5 mm) were sterilised by dripping in 70% ethanol, washed three times with PBS, and placed in flat-bottomed 24-well plates (353047, Falcon, Corning Inc., Corning, NY, USA). In our extensive experience with the FA material, sterilisation with 70% ethanol does not damage the matrix. The scaffolds were seeded with 1 × 10⁵ MC3T3-E1 subclone 4 mouse pre-osteoblasts (Lot 62044205, CRL-2593, ATCC, Manassas, VA, USA) in 50 µL medium. After seeding, the plates were incubated for 3 h at 37 °C with 5% CO₂ to allow cells to attach to the scaffolds. After the attachment incubation time, 1 mL of αMEM (Minimum Essential Medium Eagle Alpha Modification (A10490-01) with 100 U/mL penicillin/streptomycin (15140-122) and 10% foetal bovine serum (FBS, 10270-106), all from Gibco, Paisley, UK)) with or without osteogenic supplements (50 µg/mL ascorbic acid (A0278), 10 mM β-glycerophosphate (G9422), and 100 nM dexamethasone (D4902), all from Sigma-Aldrich, Dublin, Ireland) was added per well and cultured over a 28-day period at 37 °C with 5% CO₂.

### 2.8. Cell Viability by Live/Dead Assay

Seeded scaffolds (*n* = 1 per scaffold group in culture medium with/without osteogenic supplement) were assessed for cell incorporation and viability using Live/Dead cell double staining kit according to the manufacturer’s instructions (04511-1KT-F, Sigma-Aldrich, Dublin, Ireland), wherein live cells fluoresce green and dead cells fluoresce red. Briefly, on days 1, 7, 14, 21, and 28 of culture, scaffolds were washed in PBS prior to staining with the calcein-AM and propidium iodide (PI) staining solution, and then the staining procedure was performed in the dark for 30 min at 37 °C and 5% CO_2_. Live and dead cells were visualized under a laser confocal microscope (LEICA DMIRE2, Leica, Wetzlar, Germany).

### 2.9. Cell Proliferation by Alamarblue™ Assay

Seeded scaffolds (*n* = 3 per scaffold group in culture medium with/without osteogenic supplement) were transferred to fresh 24-well plates and 1 mL of alamarBlue™ (DAL1100, Invitrogen, Paisley, UK) working solution, (diluted 10× from stock solution with phenol-free Dulbecco’s modified Eagles medium (DMEM, 11880, Gibco, Paisley, UK) supplemented with 10% FBS and 1% antibiotics) was added per well, and samples were incubated at 37 °C with 5% CO_2_ for 3 h, after which each well content was transferred to a cuvette and absorbance measured at 570 nm in a uv/vis spectrophotometer (Spectronic Camspec Ltd., Garforth, UK). Absorbance at 600 nm of phenol-free DMEM was read and subtracted from the test sample absorbance to obtain the final value.

### 2.10. Cell Infiltration through the Scaffolds

Cell-seeded scaffolds (*n* = 3 per scaffold group in culture medium with/without osteogenic supplement) were fixed in 4% paraformaldehyde at days 7, 14, 21, and 28 of culture and processed for paraffin histology. Cross-sections were cut to a thickness of 4 µm and stained with haematoxylin and eosin (H&E), which stains cell nuclei purple/black, while the cytoplasm appears bright pink. Stained sections were dehydrated and cover-slipped for observation under light microscopy (Zeiss Axiophot, Zeiss, Jena, Germany; DC200 Leica digital camera and Micro-Manager 1.4 software).

### 2.11. Angiogenesis by an Ex Ovo Chorioallantoic Membrane (CAM) Assay

Pro-angiogenic potential was assessed using an ex ovo CAM assay as already described [38]. Scaffolds (PCL, FA, and PCL/FA, *n* = 4 per material) were cut into 5 mm × 5 mm square pieces, sterilised with 70% ethanol, and washed three times with PBS. Fertilized chicken eggs were incubated in an egg incubator at 38 °C with automatic rotation. Humidity was maintained between 45% and 50%. At day 3 post-incubation, the embryos were transferred to a shell-less culture system with 80–90% humidity and 38 °C incubation temperature. At day 6 of culture, scaffolds were orthotopically placed onto the developing CAMs, and the ex ovo cultures were incubated for a further 3 days, when embryos were euthanised under the British Home Office regulations by freezing at −20 °C for 15 min. An amount of 5 mL of 4% paraformaldehyde was added onto the CAM and covered for 15 min to avoid bleeding of CAM after excision. The scaffolds were then dissected along with a 5 mm perimeter of the CAM. Images were acquired using a GT Vision stereo microscope (GXM-XTL3T101, GT Vision, Wickhambrook, UK) by inverting the scaffolds to observe infiltrating blood vessels from underneath. Stereo microscope images were processed using threshold function and then converted into binary images for calculating the percentage vascular area (normalised to the area of the scaffold) using ImageJ software (version 1.51, U.S. National Institutes of Health, Bethesda, MD, USA). Bifurcation points (branch points seen in the vasculature within a given scaffold) were counted using the ImageJ “counter” function. 

### 2.12. Surgical Procedure of In Vivo Assessment of Scaffold in a Rat Cranial Defect Model

All animal experimental procedures were approved by the Animal Ethics Committee at Queensland University of Technology (approval #1700001069), in compliance with the “Australian code for the care and use of animals for scientific purposes, 2013”. 

Eighteen skeletally mature male Lewis rats (average weight = 289.6 ± 19.8 g) were operated on under general anaesthesia. The rats were individually placed into an anaesthetic induction chamber and induced with 3% isoflurane in 100% oxygen (3 L/min). General anaesthesia was then maintained with 2% isoflurane in 100% oxygen (1–1.5 L/min) via a rodent nose cone. Buprenorphine (0.01 mg/kg subcutaneously), local anaesthetic with adrenaline (2% lignocaine, 1/80,000 adrenaline subcutaneously), and meloxicam (1 mg/kg subcutaneously) were administered for pre-emptive analgesia. Postoperatively, tramadol (25 mg/L of drinking water) and buprenorphine (0.01 mg/kg) were used for analgesia. During anaesthesia, heart rate, respiration, and blood oxygen saturation (SpO_2_) were continuously monitored. Surgical antibiotic prophylaxis was administered prior to incision (cefazolin, 20 mg/kg, subcutaneously). Fronto-parietal region of the skull was prepared aseptically and draped. A 15 mm midline skin incision was then made between the ears and dissected down to the periosteum. The periosteum was then sharply divided to expose the calvarium. Two full-thickness bone defects (5 mm in diameter) were then trephined in the centre of the parietal bone using a slow speed dental drill, under continuous saline irrigation. Extreme caution was exercised to avoid over-trephination and subsequent perforation of underlying dura matter. Two scaffolds of all experimental groups: FA, PCL, and FA/PCL were then implanted into the bone defects in each rat according to the implantation plan. The skin incision was then closed in layers using absorbable monofilament suture. Animals were then administered 100% oxygen and transferred to a clean warm cage until full recovery.

All animals were carefully monitored postoperatively for adverse effects or local reactions to the implants. The rats were humanely killed with CO_2_ asphyxiation at 4 weeks or 12 weeks post-surgery. The cranial explants were retrieved using an Exakt band saw. The tissue explants were fixed in 4% paraformaldehyde (PFA) and assessed by microcomputed tomography and histology.

### 2.13. Microcomputed Tomography (µCT)

The rat cranial explants were scanned with a micro-computed tomography (µCT) on an in-house machine (μCT 40, Scanco Medical, Bruttisellen, Switzerland) at a source voltage of 55 KV and a current of 145 µA. Samples were scanned in a tube with a diameter of 19 mm and 84 mm length in 70% ethanol. This resulted in an isotropic voxel size of 10 µm. The grayscale images were evaluated by applying a region of interest at the defects with Scanco Medical software to obtain the quantification of bone volume (BV) and total volume (TV) of defects. The lower threshold of 220 was selected based on the histogram to best differentiate mineralized bone with background noise. 

### 2.14. Histology: Paraffin Embedding and Sectioning

All cranial explant samples were cut with an EXAKT saw to divide individual defects for analysis using paraffin and resin histology. One sample of each experimental group of both time points was decalcified with a rapid decalcifier (Kos Milestone microwave, ABACUS, Brisbane, Australia) at 37 °C. The demineralized samples were processed with an Excelsior ES Tissue Processor (Thermo Scientific, Scoresby, VIC, Australia) and embedded with paraffin wax at an embedding station (Thermo Scientific, Scoresby, VIC, Australia). Five-micron sections were obtained with a Leica RM2235 rotary microtome (Leica Biosystems, Nussloch, Germany).

### 2.15. Haematoxylin and Eosin Stain (H&E)

To assess tissue morphology, H&E staining was performed on the paraffin sections of the rat samples by an autostainer (Leica Autostainer XL, Leica Biosystems, Nussloch, Germany) using the lab-standard protocol. Briefly, the tissue sections were deparaffinized and rehydrated with decreasing concentrations of ethanol and washed in DI water before being stained in Mayer’s haematoxylin for 4 min. The sections were differentiated in acid alcohol for 5 sec, blued with tap water, and then stained in eosin for 1 min. After staining, the stained sections were dehydrated and mounted using Eukitt mounting medium (03989, Sigma-Aldrich, MacQuarie Park, Adelaide, Australia).

### 2.16. Immunohistochemistry (IHC)

Immunohistochemistry was carried out on paraffin sections as described previously [39,40]. Briefly, the sections were deparaffinized and rehydrated before they were incubated for 10 min with proteinase K (S302080, Dako, Sydney, Australia) for antigen retrieval. Following a 10 min incubation with 3% hydrogen peroxide solution, the slides were blocked with 2% BSA for 40 min. The sections were incubated overnight at 4 °C, with primary antibodies diluted in 2% BSA to the following concentrations: collagen type I (Col I, 1 ug/mL, Abcam, Cambridge, UK), alkaline phosphatase (ALP, 5 ug/mL, Abcam, Cambridge, UK), osteocalcin (OC, 1 ug/mL, Abcam, Cambridge, UK), CD34 (Abcam, 5 ug/mL), and von Willebrand factor (vWF, IR52761-2, ready to use, Agilent, Santa Clara, CA, USA). Mouse/rabbit IgG (Thermo Fisher Scientific, Scoresby, VIC, Australia) and 2% BSA were used as isotype control and negative control, respectively. The sections were then incubated with secondary antibody (EnVision + Dual Link System-HRP rabbit/mouse, Dako K4061, Agilent, Santa Clara, CA, USA) for 45 min at RT. For chromogen development, the antigens were detected with diaminobenzidine (DAB) (DAKO K3468, Agilent, Santa Clara, CA, USA). All sections were counterstained with Mayer’s haematoxylin, and the slides were mounted for imaging. The samples slides were scanned with a 3D Histech Scan II Fluorescence/Brightfield Slide Scanner (3DHISTECH, Budapest, Hungary). In each of the IHC stained sections of ALP, OC, CD34, and vWF, 6 random areas were selected within the defect area for quantification with Fiji (ImageJ) software using a published protocol [41].

### 2.17. Histology: Resin Embedding and Sectioning

Samples for resin embedding were dehydrated with increasing grades of ethanol and then infiltrated and embedded in Technovit 9100 methyl methacrylate system (Kulzer GmbH, Wehrheim, Germany) without decalcification. A total of 8 µm thin sections were obtained via a heavy-duty sledge microtome (Polycut-S, Reichert-Jung, International Medical Equipment, Miami, FL, USA), and thicker 50 µm ground sections were obtained using an EXAKT cutting and grinding system. 

### 2.18. Von Kossa Stain

The 8 µm thin resin sections were stained with von Kossa stain as described previously [39]. Briefly, deplasticised resin sections were rehydrated in decreasing grades of ethanol and washed with distilled water before 5 min incubation with 1% *w*/*v* silver nitrite solution. The colour development was performed with 5% *w*/*v* sodium carbonate–formaldehyde solution for 2 min. The sections were counterstained with MacNeal’s tetrachrome solution (Dorn and Hart Microedge Inc., Villa Park, IL, USA), dehydrated in ethanol, cleared in xylene, and cover-slipped for imaging.

### 2.19. Goldner’s Trichrome Stain

The thick ground sections were stained with a Goldner’s trichrome protocol as published previously [42]. Briefly, the sections were stained with Weigert’s haematoxylin (Merck, Bayswater, VIC, Australia) for 25 min, then washed and immersed in acid Fuchsin–Ponceau working solution Fuchsin (Merck, Bayswater, VIC, Australia) for 10 min. Following washes in 1% acetic acid, the sections were stained with tungstophosphoric acid–orange G Fuchsin solution (Merck, Bayswater, Australia) for 20 min and light green solution for 15 min. After air drying, the sections were cleared in xylene and mounted for imaging. 

### 2.20. Statistical Analysis

In vitro results were statistically analysed by one-way ANOVA with a Holm–Sidak post hoc analysis using Sigma Stat 3.5 software (Systat Software Inc., San Jose, CA, USA). In vivo statistical tests were performed by two-way ANOVA with GraphPad Prism 8 software (GraphPad Software Inc., San Diego CA, USA). The BV/TV data obtained by µCT were analysed with a mixed error-component model, and the IHC quantification data were analysed with two-way ANOVA. A *p* < 0.05 was considered a significant result.

## 3. Results

### 3.1. PCL Surface Treatment

**Figure 2:**
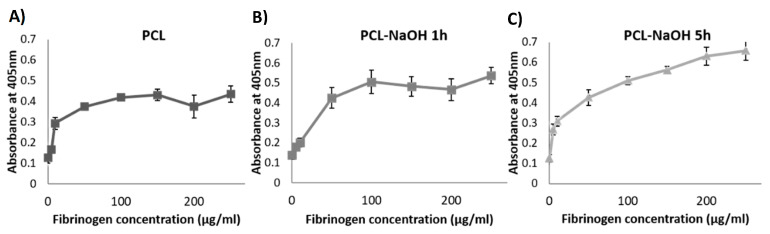
Results from the immuno-based assay on fibrinogen adsorption on the three different PCL surfaces tested: (**A**) native PCL, (**B**) alkaline etching with 5 M NaOH for 1 h, and (**C**) 5 h. Results show mean ± standard error of the mean.

Results from the immuno-based assay showed that the surface treated with 5 M NaOH for 5 h could adsorb higher amounts of fibrinogen in comparison with the other surfaces (Figure 2). Therefore, this surface was chosen to fabricate the PCL/FA composites. 

### 3.2. SEM 

**Figure 3:**
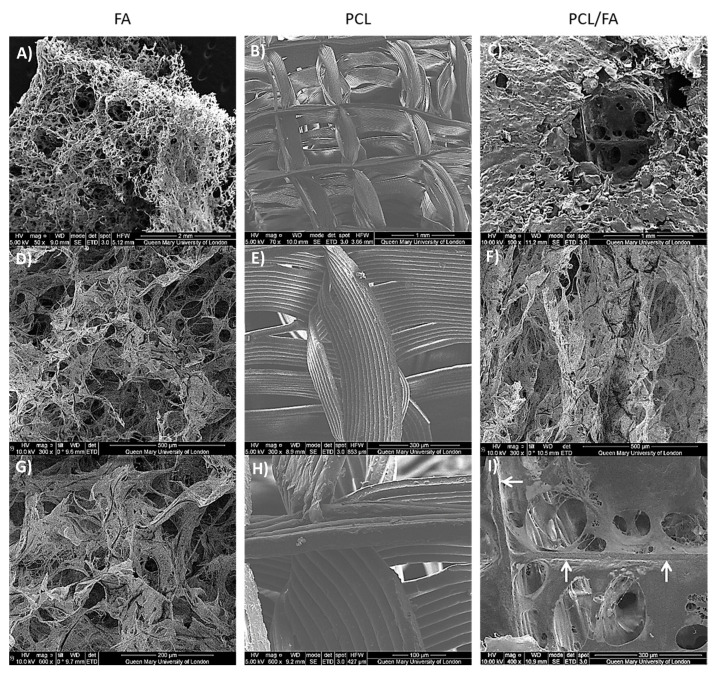
Scaffold morphologies shown by SEM imaging: (**A**,**D**,**G**) FA, (**B**,**E**,**H**) PCL, and (**C**,**F**,**I**) PCL/FA. Images in **A**–**C** are at 50×, 70× and 100× respectively, **D**–**F** are at 300×, and **G**–**I** are at 600× (**G**,**H**) and 400× (**I**). White arrows in image I show binding of FA matrix to the PCL fibres.

SEM images of PCL/FA composites (Figure 3) showed that the open pore structure of the FA matrix was maintained in the PCL/FA composites. Direct binding of the FA material with the PCL fibres could be observed (Figure 3 white arrows).

### 3.3. ATR-FTIR

**Figure 4:**
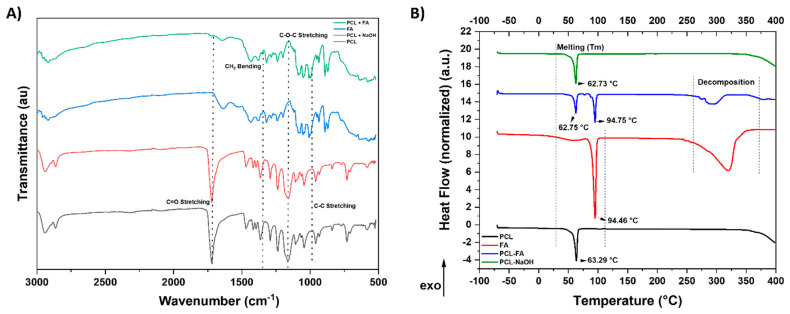
(**A**) ATR-FTIR characterisation spectra of PCL (black), PCL with NaOH treatment (red), FA (blue), and PCL/FA (green). (**B**) DSC profile of scaffolds. Melting points are indicated on the graph.

Figure 4A shows ATR-FTIR results on the different scaffolds. As indicated on the graph, the main peaks found were C=O stretching, CH_2_ bending, C–O–C stretching, and C–C stretching. PCL and PCL treated with NaOH had identical spectra, while the spectra of FA and PCL/FA were very similar.

### 3.4. DSC

Figure 4B shows DSC results of the pure PCL and fibrin/alginate (FA) and composites of PCL-FA and PCL-NaOH (respectively from the bottom line). The melting points (T_m_) of all samples and decomposition area of FA-containing samples are stated in the graph. Crystallinity (Xc) of the composites is shown in Table 1, and as seen, there is a significant decrease in crystallinity of PCL-FA (48.37%) composites compared with pure PCL (91.47%) and PCL-NaOH (86.18%).

### 3.5. Cell Viability by Live/Dead Assay

**Figure 5:**
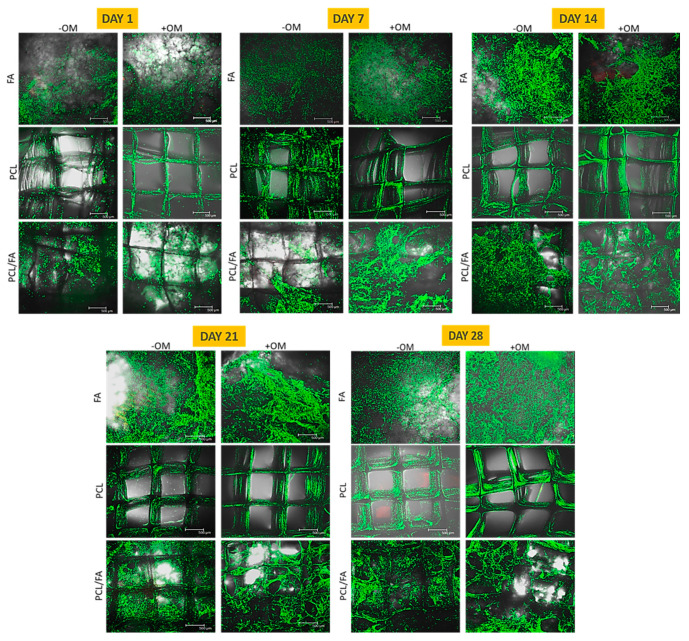
LIVE/DEAD assay results showing viable cells (green fluorescence) on all materials at all time points. Scale bar = 500 μm.

Results show that cells were viable on all scaffolds over the culture period (Figure 5). At early time points (days 1 and 7), cells preferentially bound to the FA component of the PCL/FA composites, suggesting that the FA material may promote initial cell attachment. At later time points (days 21 and 28), it could be observed that cells colonised the PCL fibres of the PCL/FA composites. Moreover, confocal images also showed different cellular morphology between samples cultures under standard conditions, without osteogenic supplements (-OM), and samples cultured under osteogenic conditions (+OM): cells cultured in osteogenic medium tended to form aggregates over time, while cells cultured under standard conditions remained separate and more individually dispersed. This would be indicative of active osteogenic differentiation under osteogenic conditions. 

### 3.6. Cell Proliferation by Alamarblue™ Assay

**Figure 6:**
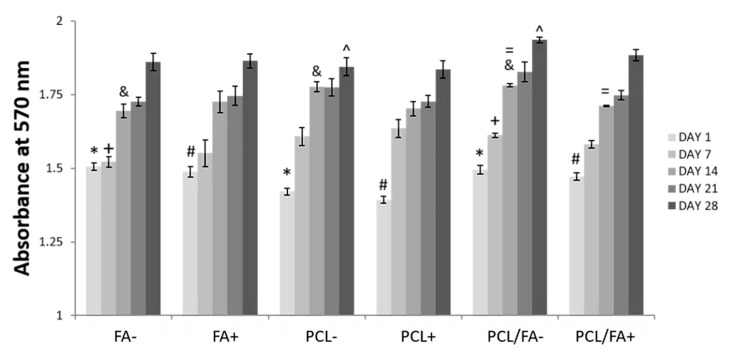
AlamarBlue™ assay results for MC3T3-E1 osteoprogenitor cells seeded on FA, PCL, and PCL/FA scaffolds and cultured in either standard medium (−) or osteogenic medium (+) for 28 days. Results show mean ± standard error of the mean. * Both FA− and PCL/FA− significantly higher than PCL− at day 1. # Both FA+ and PCL/FA+ significantly higher than PCL+ at day 1. + PCL/FA− significantly higher than FA− at day 7. & Both PCL− and PCL/FA− significantly higher than FA− at day 14. = PCL/FA− significantly higher than PCL/FA+ at day 14. ^ PCL/FA− significantly higher than PCL− at day 28. Statistical significance when *p* < 0.05.

Cells were able to proliferate on the different scaffolds in both osteogenic and standard media for the duration of the study (Figure 6). A number of statistical significances were found, which can be seen in Figure 6. Most notably, cells on FA and PCL/FA scaffolds showed significantly higher metabolic activity than on PCL scaffolds on day 1, both in standard and osteogenic media, suggesting that FA may promote early cell attachment onto the scaffolds, as observed on the LIVE/DEAD assay. On day 28, metabolic activity for the PCL/FA composite was significantly higher than in PCL when cultured under standard conditions, suggesting that combining both biomaterials (PCL and FA) results in a composite scaffold with superior properties in terms of cell growth.

### 3.7. Angiogenesis by an Ex Ovo CAM Assay

**Figure 7:**
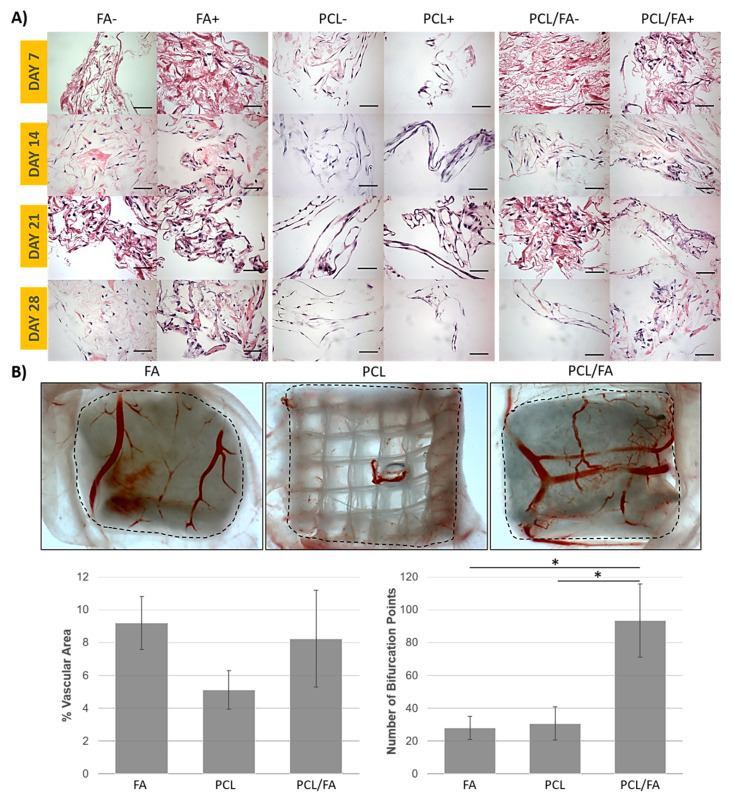
(**A**) Cell infiltration results by paraffin histological processing and H&E staining of MC3T3-E1 osteoprogenitor cells seeded on FA, PCL, and PCL/FA scaffolds and cultured in either standard (−) or osteogenic medium (+) for 28 days. Cell nuclei are stained purple, while FA matrix is stained pink. Scale bar = 50 μm. (**B**) Results from the ex ovo CAM assay. Top, representative stereomicroscopy images of FA, PCL, and PCAL/FA scaffolds (5 mm × 5 mm pieces; dashed line indicates border of scaffold). Bottom, percentage of vascular area and number of bifurcation points (* *p* < 0.05: PCL/FA has a significantly higher number of bifurcation points compared with both PCL and FA groups). Results show mean ± standard error of the mean.

Results from the CAM assay show blood vessel infiltration into all scaffolds, although macroscopically more blood vessels were seen on FA and PCL/FA scaffolds than on PCL ones (Figure 7B). Quantification of percentage of vascular area showed a positive trend for FA and PCL/FA scaffolds, although results were not significant. In terms of number of bifurcation points, a significantly higher number was observed for PCL/FA composite scaffolds compared with both FA and PCL controls.

### 3.8. In Vivo Bone Regeneration by µCT

**Figure 8:**
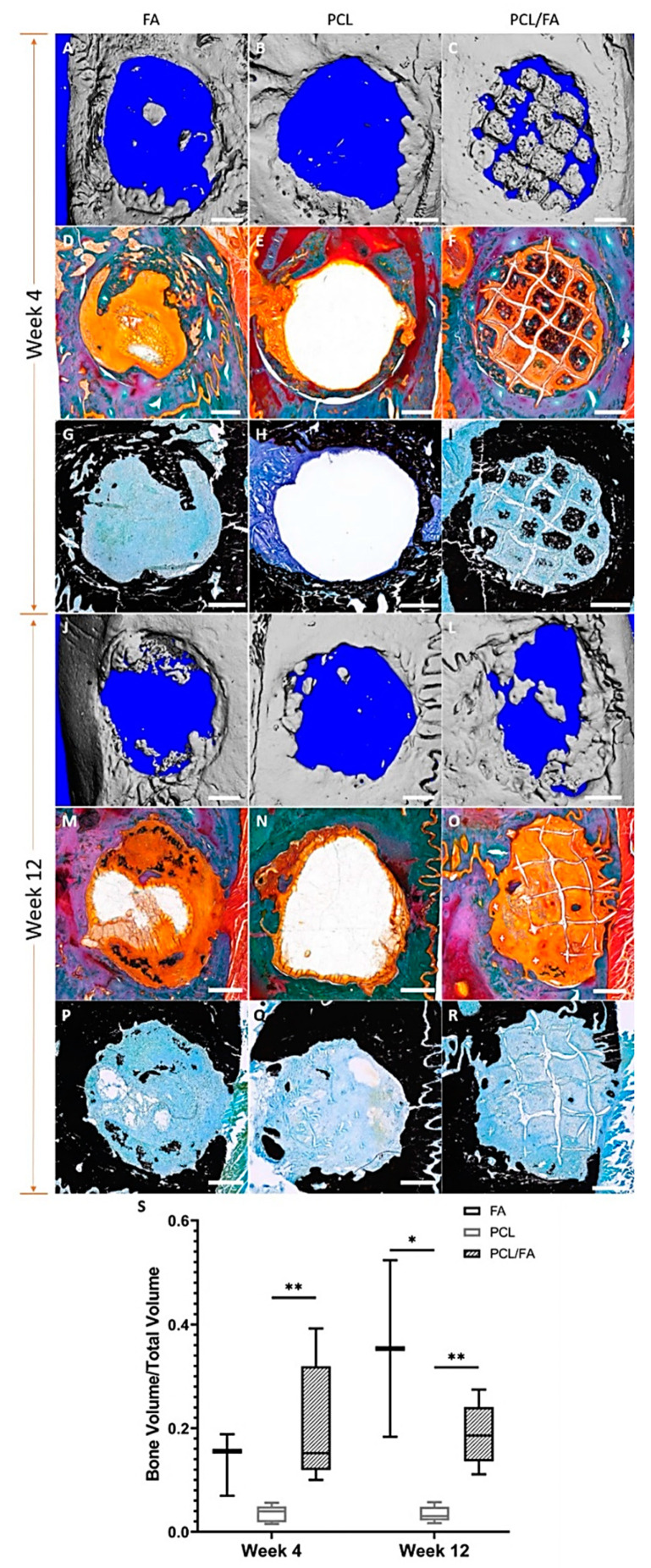
μCT and histological assessment of cranial defects after 4 and 12 weeks of scaffold implantation. Representative 3D reconstruction of mineralized bone tissues is shown (grey colour) at 4-week (**A**–**C**) and 12-week (**J**–**L**) time points. The images show the volume of host bone around the defect and regenerated bone in the defect. The μCT quantification of regenerated bone volume to total defect volume ratio is shown at the bottom (**S**). The centre line denotes the median value, while the box contains the 25th to 75th percentile of the dataset. The whiskers mark the min/max values. * and ** indicate a *p* < 0.05. In the Goldner’s trichrome staining images at the 4-week time point (**D**–**F**) and 12-week time point (**M**–**O**), the green/blue colour indicates mineralized bone matrix, and orange colour shows connective tissues formed of mainly collagen fibres. In the von Kossa stained images at the 4-week time point (**G**–**I**) and 12-week time point (**P**–**R**), the mineralized bone matrix stains black, and blue counterstain shows connective tissues. Scale bar = 1 mm.

At the 4-week timepoint, µCT analysis of bone regeneration within the cranial defects showed increased bone volume in both the FA and PCL/FA scaffold groups compared with PCL, and the increase was significantly higher in the PCL/FA group. Noticeably, islands of bone tissues were found across the defect in the PCL/FA group, while the bone regeneration happened adjacent to the host tissue, as shown in Figure 8A,C, respectively. At the 12-week timepoint, both FA and PCL/FA scaffold groups showed significantly higher bone volume compared with the PCL group. The regenerated bone tissues were predominantly found at the interface to the host tissues in both the FA and PCL/FA groups, as shown in Figure 8J,L.

### 3.9. Goldner’s Trichrome and Von Kossa Stains

Goldner’s trichrome stain (Figure 8D–F,M–O) differentiates mineralized bone matrix from surrounding connective tissues: bone matrix coloured between green and blue, collagen fibres stain orange, and soft connective tissues in red. Von Kossa stain (Figure 8 G–I,P–R) identifies mineralized tissues by staining them black. Both stains on resin sections showed mineralized bone matrix in close agreement with µCT images. In the PCL/FA group, bone tissues were found in the defect between the scaffold fibres after 4 weeks of implantation (Figure 8F,I). The space taken by the PCL fibres could be clearly seen between the islands of newly formed bones. Within each bone island, we could also see the gap of spaces occupied by the FA component. At the 12-week timepoint, small areas of bone islands could be seen in the PCL/FA scaffold group, but most of the regenerated bone was found integrated into the host tissue (Figure 8O,R).

### 3.10. H&E Stain and Immunohistochemistry

**Figure 9:**
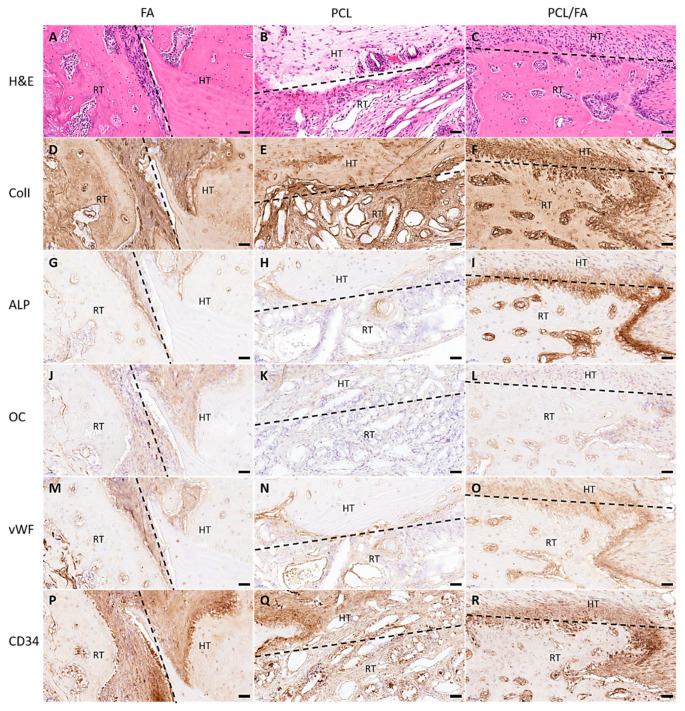
H&E and IHC staining of rat cranial tissues at the host tissue (HT)/regenerated tissue (RT) interface at the 4-week time point. The interfaces are indicated by the dashed lines. H&E staining images showing the tissue morphology (**A**–**C**) and IHC staining of rat cranial tissues showing the positive markers found on the surface of tissues: ColI (**D**–**F**), ALP (**G**–**I**), OC (**J**–**L**), vWF (**M**–**O**), and CD34 (**P**–**R**). Scale bar = 50 μm. ALP, alkaline phosphatase; ColI, collagen type I; OC, osteocalcin; vWF, von Willebrand factor.

**Figure 10:**
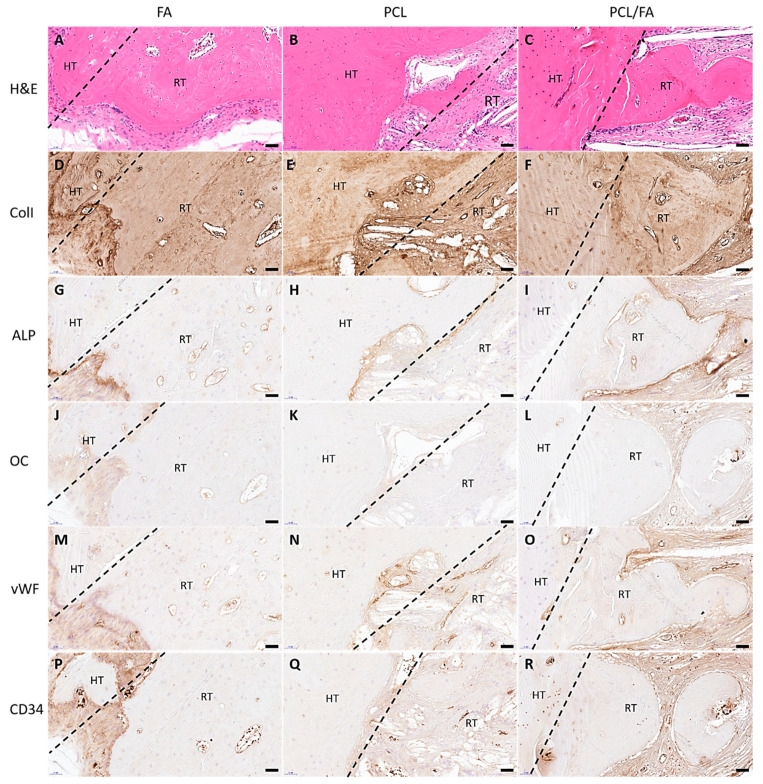
H&E and IHC staining of rat cranial tissues at the host tissue (HT)/regenerated tissue (RT) interface at the 12-week time point. The interfaces are indicated by the dashed lines. H&E staining images showing the tissue morphology (**A**–**C**) and IHC staining of rat cranial tissues showing the positive markers found on the surface of tissues: ColI (**D**–**F**), ALP (**G**–**I**), OC (**J**–**L**), vWF (**M**–**O**), and CD34 (**P**–**R**). Scale bar = 50 μm. ALP, alkaline phosphatase; ColI, collagen type I; OC, osteocalcin; vWF, von Willebrand factor.

The morphology of the tissues in and around the defects was revealed by H&E stain by staining the cell nuclei blue and cytoplasm pink. After 4 weeks and 12 weeks, mature bone tissues were found in the defects in the FA and PCL/FA groups (Figure 9A,C and Figure 10A,C). The regenerated tissues in the PCL control group were found to be mostly soft connective tissues (Figure 9B and Figure 10B).

**Figure 11:**
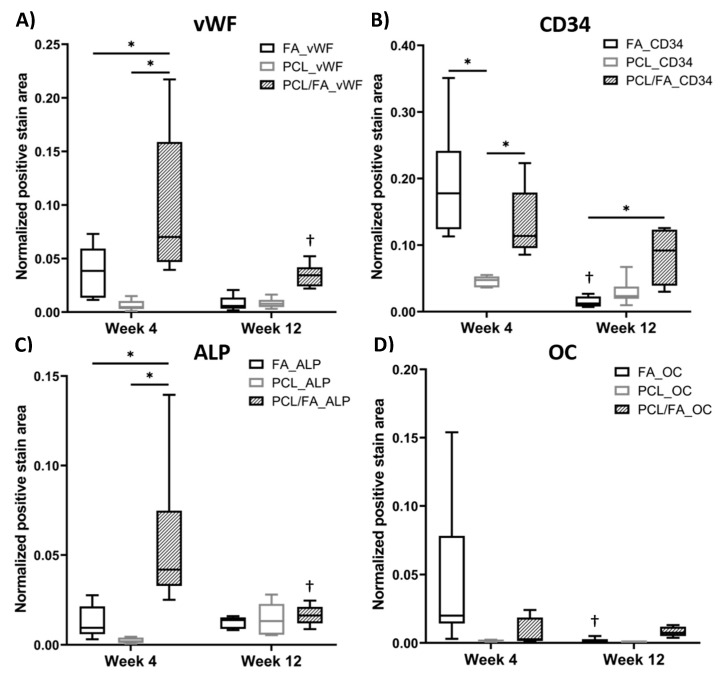
Quantitative analysis of IHC investigating the positive stain areas of (**A**) vWF, (**B**) CD34, (**C**) ALP, and (**D**) OC within the defect site. The centre line denotes the median value, while the box contains the 25th to 75th percentile of the dataset. The whiskers mark the min/max values. * and † indicate a *p* < 0.05. * shows significant difference between groups at the ends of bars, † shows significant difference between the same scaffold group at 4-week and 12-week timepoints (vWF and ALP: PCL/FA_4 week and PCL/FA_12 week; CD34 and OC: FA_4 week and FA_12 week).

IHC was used to show the distribution of angiogenic markers vWF and CD34, osteogenic markers ALP and OC, and bone matrix marker ColI in the regenerated tissues. In Figure 9, both angiogenic and osteogenic makers show higher intensity in PCL/FA (I, L, O, R) and FA (G, J, M, P) groups compared with the PCL control group (H, K, N, Q) after 4 weeks of implantation. Such difference was not noticed in the 12-week samples, as shown in Figure 10. In Figure 11, the quantification of the IHC stain showed significantly enhanced angiogenic markers in the PCL/FA groups at both timepoints and showed significantly enhanced osteogenic markers at the 4-week timepoint. More specifically, the PCL/FA scaffold group showed higher blood vessel formation, indicated by endothelial cell markers of vWF and CD34. The FA group showed enhanced blood vessel formation compared with the PCL controls. Similarly, the PCL/FA group also showed a higher amount of ALP, with the difference being significant at the 4-week time point. Both FA and PCL/FA groups showed an increased amount of OC, but no significant difference was found. The PCL/FA group showed significantly decreased vWF and ALP markers at the 12-week timepoint when compared with the 4-week timepoint. The FA group showed significantly decreased CD34 and OC markers at the 12-week timepoint compared with the 4-week timepoint.

## 4. Discussion

Limitations of current graft materials used for treating bone defects make necessary the development of new biomaterials [1]. By developing a composite biomaterial of pro-angiogenic FA and robust PCL, their beneficial properties would be combined. Moreover, the composite would be porous for bone ingrowth.

Initially, different surface treatments for PCL were investigated, as previous research had shown that the FA matrix only binds to hydrophilic surfaces [34,36] and that PCL is a hydrophobic polymer [11]. The surface treated with 5M NaOH for 5 h could adsorb higher amounts of fibrinogen in comparison with the other surfaces (Figure 2), and therefore it was chosen to fabricate the PCL/FA composites. PCL surface modification with NaOH etching reduces hydrophobicity and increases surface roughness of the PCL fibres via hydrolysis and formation of carboxyl groups on the fibre surfaces [18,22]. It has already been shown that the hydrolysis process with NaOH does not induce deformation of the fibres and that they retain their diameter [18]. 

Using a foam-based manufacturing process, results show that composites integrating both materials were obtained, where thick PCL fibres acted as a backbone throughout which the FA fibres formed a mesh (Figure 3). Physicochemical characterisation of the scaffold was assessed by ATR-FTIR and DSC. Surface modification of PCL via hydrolysis did not really change the FTIR spectrum since the newly introduced carboxyl groups (-COOH) would be shown in the C=O stretching area of the spectrum, which was already present in the untreated PCL. The spectrum of PCL/FA was identical to the FA one, indicating that the FA component completely covered the PCL fibres. Regarding the DSC results, Kim and Kim [43] reported that alginate addition into PCL decreased crystallinity of the composites, which might be attributed to the hydrogen bond interaction between the carbonyl groups of the PCL and the OH groups in the alginate. Although further mechanical characterization is required for our scaffolds, from the degree of crystallinity analysis it can be estimated that the mechanical properties of the PCL/FA composites may show a decrease in the elastic modulus compared with PCL since there is a monotonic correlation between crystallinity and Young’s modulus [44].

MC3T3-E1 osteoprogenitor cells remained viable and proliferated over the culture period under both standard and osteogenic conditions on all scaffolds. The FA component of the composites may promote initial cell attachment. Good cellular integration with both components of the composite was observed. This is important since, after implantation, autologous cells will eventually come in contact with both FA and PCL fibres. Furthermore, von Kossa stain results indicate that cells differentiated along the osteogenic pathway on the scaffolds when cultured under osteogenic conditions (results not shown), as already demonstrated by numerous studies [21,22].

Bone is a highly vascularised tissue, and therefore, any bone regeneration strategy should consider angiogenesis [23]. Assessment of pro-angiogenic potential using an ex ovo CAM assay [38] showed blood vessels infiltration into all the scaffolds, which was expected since all the scaffolds are highly porous. The percentage of vascular area was lowest for PC scaffolds. Both FA and PCL/FA contain fibrin, which is a pro-angiogenic natural polymer [24]. Of note, a significantly higher number of bifurcation points for PCL/FA compared with both PCL and FA was seen. Bifurcation of blood vessels is important for the formation of a capillary plexus allowing delivery of nutrients and oxygen and removal of metabolites, thus ensuring vascularisation throughout the injured area [22].

A proper blood supply is essential for bone regeneration. Vasculature plays a pivotal role in nutrient supply, oxygen supply, and metabolite removal, maintaining bone cell viability as these blood vessels provide specific growth factors that stimulate mesenchymal stem cells into osteoblasts [45,46]. FA sheet scaffolds have been designed and applied to heal skin wounds for their angiogenic abilities [33], and here it was the first time FA was applied to heal bone defects. To further test the bone regenerative capacity of the PCL/FA composite scaffolds, we performed pre-clinical assessments by implanting the scaffolds into critical-sized bone defects in a rat cranial defect model [47,48]. The in vivo implantation showed no adverse effect from these scaffolds, and post-operation assessment showed interesting results worth bringing to the attention of fellow scientists. 

At the timepoints of assessment (4 and 12 weeks), the PCL/FA and FA groups outperformed the PCL control group, showing higher volumes of bone regeneration, with a significant difference between PCL/FA and PCL. The PCL/FA group showed the best osteogenic capacity, as new bone was found in the pores of scaffolds within the defect at 4 weeks, as shown by µCT (Figure 8C). This indicates that the bone regeneration started with mesenchymal cells attaching onto the PCL fibres and FA matrix and proliferated into the centre. The mineralized bone matrix identified by Goldner’s trichrome and von Kossa stains showed congruent results with the high-density objects found by µCT scans, as shown in Figure 8. The histology results also indicated the regenerated bone matrix being ordered and mature within the defects. The FA scaffold works as a template for cell and tissue attachment by providing large surface areas and more importantly as a reservoir of blood and growth factors at the initial stage of healing that is beneficial to the process of bone healing. The PCL scaffold provides additional structural support to the cells attached and cell matrix produced. When the two materials were combined, the PCL/FA composite scaffolds were able to better promote blood vessel formation, as shown by the significantly higher vWF/CD34-positive areas compared with the FA and PCL groups. This was consistent with the in vitro CAM study. We postulate a cascade of events happened due to enhanced vasculature formation starting with the upregulation of ALP and OC, which are important markers of osteoblast differentiation. The osteoblasts are responsible for new bone matrix production [49], and they would differentiate into osteocytes as the matrix mineralizes, eventually resulting in the osteocyte residing within the mineralized bone matrix. The quantification of the ALP marker showed elevated osteoblast activity, and again, the PCL/FA scaffolds showed significantly higher ALP than other two groups. The FA scaffolds were also able to promote angiogenesis and had shown higher bone volume within the defects where they were implanted. However, when compared with the PCL/FA composite scaffolds, the FA scaffolds lacked the additional mechanical reinforcement from PCL fibres to facilitate cell attachment and proliferation across the defect areas, thus they could not achieve the bridging of defects. 

To assess the long-term bone healing efficacy of the FA and PCL/FA scaffolds, we extended our observation period to 12 weeks. This assessment time point was intentionally selected to allow the complete degradation of FA scaffolds, which takes approximately 42 days according to our previous in vitro degradation data [32] and in vivo observations (results not published). Our in vitro investigation of FA degradation used the serine protease trypsin to mimic the degradation phase of the wound healing environment, where proteases play a key role [50]. Our data showed that the FA scaffold degrades via bulk degradation, with the protease penetrating the entire scaffold bulk, thereby causing hydrolysis throughout the entire polymer network, which eventually breaks down into smaller pieces [16]. As already mentioned, it took 42 days (6 weeks) for the FA scaffolds to completely degrade in vitro, which correlates with the observations made in a porcine model, where the implanted scaffolds degraded within 5–6 weeks (results not published). Therefore, at the 12-week timepoint of this study, the FA scaffolds and FA component of the PCL/FA scaffolds would have been long degraded. At the 12-week timepoint, the µCT results show that both the PCL/FA and FA groups promoted significantly higher bone volumes compared with the PCL group. However, the increase in bone volume was not significantly higher when compared with the 4-week timepoint in any of the scaffold groups. When we looked at the quantification results of vascularization and osteoblast differentiation markers with histology, a decrease that was significant in some cases was found in the FA and PCL/FA groups at the 12-week timepoint. The decreased osteogenic capacity compared with the 4-week timepoint was hypothesised to be due to the mismatch between the degradation rate of FA membrane and the bone formation rate in the rat cranium. Homma et al. investigated the subcritical-sized defects (3.8 mm in diameter) repair in rat cranium, and a full bridging of the defects was found at 24 weeks post-surgery [51]. Based on these data, the degradation rate of the FA scaffold is considerably faster than the rate at which rat cranial bone regenerates. The fast degradation of FA scaffolds did not allow sufficient time for the new bone to form and integrate with the host bone around the defects. The bone tissues formed on the FA scaffolds would have lost their attachment surface and mechanical support around 6 weeks post operation. Furthermore, bone remodelling is an active component in the whole bone regeneration process, which is driven by the interaction of osteoblasts, osteocytes, and osteoclasts [31,33,52]. Upon implantation, the primary host response to a biomaterial is the formation of haematoma and the activation of the immune system, followed by bone formation which occurs after large blood vessel infiltration into the scaffold at approximately 4 weeks after implantation [31]. Then, the newly formed woven bone undergoes remodelling to form lamellar bone, which may take 6 months or longer [31]. Therefore, the remodelling process upon implantation of the scaffolds would have started around the time the FA had been completely degraded and before the newly formed bone had integrated with the host tissue. The authors propose that the non-supported bone tissues formed in the defects had been digested by the osteoclasts during the remodelling process. In the light of this significant finding, the future design of scaffolds would require adjustment of the FA degradation rate to accommodate the rate of bone matrix formation and integration with host tissues.

In bone tissue engineering, fibrin and its composites have been investigated predominantly as a delivery vehicle of stem cells [53,54,55] and/or growth factors [56,57,58]. Osteoinductive capacity is regarded as one of the most important properties of a viable bone scaffold [59]; therefore, the materials and design of a typical scaffold for bone tissue engineering should emphasize the promotion of osteogenesis (osteoblast differentiation and proliferation) by incorporating osteogenic factors. Nevertheless, the important bone regeneration feature of vascularisation is often neglected in the design of new and effective bone graft substitutes. In this study, we introduced a new strategy to target bone tissue repair using FA as a component of composite bone scaffolds due to its angiogenic capacity. Our results show enhanced early bone formation in the FA scaffold groups, without encapsulation of pre-cultured cells or growth factor supplements, especially when the FA matrix was reinforced with MEW PCL scaffolds. However, for long-term bone regeneration, tuning of FA degradation to ensure syncing with new bone formation is likely necessary. Strategies to achieve this would involve optimization of protein concentration, degree of chemical crosslinking, or inclusion of a third component into the FA matrix.

## 5. Conclusions

We introduced a novel pro-angiogenic and biodegradable PCL/FA polymer composite scaffold for bone regeneration. This system combines an FA matrix with MEW PCL scaffolds. The PCL/FA scaffolds were found to be non-toxic to cells and promoted a significantly higher number of blood vessel bifurcations compared with FA and PCL controls. We then further investigated the osteogenic capacity of these scaffolds in a preclinical rat cranial defect model to test whether the enhanced angiogenic capacity of PCL/FA could lead to faster and better bone defect healing. µCT imaging showed increased bone regeneration in the defects treated with PCL/FA scaffolds after 4 weeks of implantation compared with PCL and FA groups. This observation was confirmed by histology, where mature bone matrix was found at the host bone/new bone interface and within the defects. Further analysis by IHC showed the PCL/FA scaffolds stimulated the formation of a higher number of blood vessels compared with the other groups, especially the PCL group. The enhanced blood vessel formation led to a significantly increased number of osteoblasts, as shown by the markers ALP and OC. Although our results show fast early bone formation at 4 weeks, this was not extrapolated to the 12-week time point. We therefore suggest that the FA component degradation rate would need adjustment before translation into clinics as implants for effective bone defect healing or applied as an interface for fast osseointegration of other medical devices.

## Figures and Tables

**Table 1:** DSC analysis results of scaffold materials showing onset temperature, peak melting temperature (Tm), enthalpy (ΔH), and crystallinity (Xc, %). Xc values are normalised by 100% crystallinity of the same polymer.

Material	Onset °C	Tm Peak °C	Enthalpy (ΔH) J/g	Crystallinity (Xc) %
PCL	56.44	63.29	127.64	91.47
PCL–NaOH	58.07	62.73	120.25	86.18
PCL/FA	59.22	62.75	67.49	48.37

## Data Availability

The raw data required to reproduce these findings also forms part of an ongoing study, but they are available to download on request.
